# Overexpression of stathmin promotes metastasis and growth of malignant solid tumors: a systemic review and meta-analysis

**DOI:** 10.18632/oncotarget.12982

**Published:** 2016-10-27

**Authors:** Rong Biaoxue, Liu Hua, Gao Wenlong, Yang Shuanying

**Affiliations:** ^1^ Department of Respiratory Medicine, First Affiliated Hospital, Xi'an Medical University, Xi'an, China; ^2^ Department of Respiratory Medicine, Gansu Provincial Hospital, Lanzhou, China; ^3^ Department of Statistics and Epidemiology, Medical College, Lanzhou University, Lanzhou, China; ^4^ Department of Respiratory Medicine, Second Affiliated Hospital, Xi'an Jiaotong University, Xi'an, China

**Keywords:** stathmin, tumors, immunohistochemistry, meta-analysis, diagnosis

## Abstract

Stathmin has been investigated to be involved in development and progress of malignant tumors. This study was to clarify the relationship between expression of stathmin and tumors and assess its clinical significance. We identified 25 studies with a total of 3,571 individuals from the electronic bibliographic databases and strictly evaluated the quality and heterogeneity of included studies. We analysed the relationship between expression of stathmin and clinical characteristics by the fixed-effects and random-effects of meta-analysis and constructed a summary receiver-operator characteristic curve to estimate the test characteristics. The results showed that patients with cancer displayed a higher stathmin expression than those of non-cancer individuals (OR, 0.31), and overexpression of stathmin correlated with tumor cell differentiation (OR, 0.73), lymph node invasion (OR, 0.80) and high TNM stage (OR, 0.67). The pooled sensitivity of stathmin for distinguishing malignant tumors was 0.73 and the specificity was 0.77. The maximum balance joint for sensitivity and specificity (the *Q*-value) was 0.7566 and the area under the curve (AUC) was 0.8234. In conclusion, these results showed that overexpression of stathmin intimately correlated with malignant behavior of tumors, suggesting it could be a risk factor of malignant tumors. Stathmin had great sensitivity and specificity indicated it should be a significant molecular biomarker for malignant tumors.

## INTRODUCTION

With increasing incidence and mortality, cancer has become a leading cause of death and major public health problem all over the world. It is reported that a total of 1,658,370 new cancer cases and 589,430 cancer deaths are projected to occur in the United States in 2015 [1]. Another investigation from China reports that an estimated 4,292,000 new cancer cases and 2,814,000 cancer deaths would occur in China in 2015 [2]. Medical studies suggest that understanding the molecular mechanism of tumors is very critical for improving the diagnosis and treatment. Especially, the level of some certain protein expression is associated with the prognosis and treatment of malignant tumors [3]. Therefore, it is very common and urgent medical problem to disclose new gene and protein expression level and molecular mechanism.

Stathmin (also known as Op18, p18, p19, stathmin 1 or metablastin), is upregulated in a variety of cancers and correlates with cell proliferation and migration of cancers, especially in malignant solid tumors [4, 5]. The main function of stathmin seems to be a major cytosolic phosphoprotein that regulates microtubule dynamics by preventing tubulin polymerization and promoting microtubule destabilization [6]. Recent studies support a role for stathmin in the growth regulation of malignant tumor cells and indicate it is involved in malignant biological behavior of cancers [4, 7–13]. Thus, stathmin may be an attractive molecular biomarker and target for the diagnosis and treatment of malignant tumors. We aimed to review the evidence in the literature to date in order to (1) disclose the expression patterns of stathmin in malignant tumors, (2) clarify the relationship between stathmin expression and malignant tumors, and (3) evaluate the clinical value of stathmin in diagnosing and monitoring of malignant tumors.

## RESULTS

### Searching process of literature

**Figure 1:**
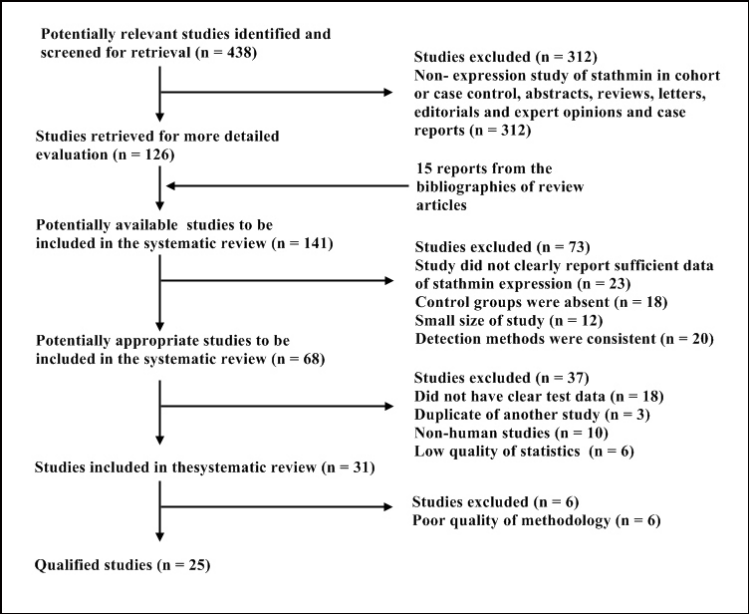
Selection of studies for review Studies were retrieved from the electronic bibliographic databases such as PubMed, Embase, Cochrane Library and SCI database.

At first, a total of 438 studies regarding stathmin expression and malignant tumors were noted from the electronic bibliographic databases. Among these studies, 126 studies seemed to be eligible, and the other 15 reports were added from the bibliographies of some articles. Of these 141 articles, we had to exclude 37 studies because of some following reasons including unclear data, duplication of data, non-human studies; and confused statistics analysis. For the rest of the 31 studies, we removed six studies finally because the low quality of methodology. Ultimately, 25 studies were included with a total of 3,571 individuals. A flow chart showing the selection of references for meta-analysis is shown in Figure 1.

### Description of studies

Table 1 listed the detailed characteristics and results of eligible studies. Of included studies, the sizes of studies ranged from 54 [14] to 323 [15] patients. The source of the malignant tumor included colorectal cancer (CRC) [15–17], pancreatic ductal adenocarcinoma (PDAC) [14, 18], hepatocellular carcinoma (HCC) [19, 20], gastric cancer (GC) [9, 21–23], esophageal carcinoma (ESCC) [24, 25], extrahepatic cholangiocarcinoma (EHCC) [6], upper urinary tract urothelial carcinoma (UUT-UC) [26], cervical carcinomas (CCA) [27], lung cancer (LC) [10, 12], endometrial carcinoma (EC) [13], nasopharyngeal carcinoma (NPC) [28], breast carcinoma (BCA) [29, 30], oral squamous-cell carcinoma (OSCC) [31], glioma [32], and cutaneous squamous cell carcinoma (CSCC) [8]. In these studies, most of investigations were in East Asia, including China, Japan, Singapore and Taiwan. Two Europe studies were in Slovenia [30] and France [29], and one study was in America [15] (Table 2).

**Table 1:** Individual characteristics and results of eligible studies

Source of tumor	Authors	Year	Control/cancer	Age (years)	Male/female (N)	Tumor stage (N)		Tumor differentiation (N)		Lymphatic Metastasis (N)	
				Cancer	Cancer	I-II	III-IV	Well to moderate	Poor	Yes	No
CRC	Ogino S [15]	2009	0/546	66.1 ± 8.5	323/223	319	227	499	31	−	−
	Tan HT [16]	2011	0/324	−	154/170	142	182	288	36	−	−
	Zheng P [17]	2009	0/149	−	96/53	73	69	39	22	70	75
PDAC	Li J [18]	2015	40/87	60.7	54/33	57	30	54	33	47	70
	Lu Y [14]	2014	0/54	−	28/26	39	15	36	18	15	39
HCC	Yuan RH [20]	2006	21/21	55.8	125/31	71	85	37	119	−	−
	Gan L [19]	2010	72/120	−	−	−	−	−	−	−	−
GC	Ke B [23]	2013	40/40	53.8	138/72	71	139	85	125	121	89
	Jeon TY [21]	2010	0/226	−	42/51	58	36	−	−	24	70
	Liu X [9]	2015	56/56	−	31/25	14	42	27	29	40	12
	Kang W [22]	2012	0/111	−	77/34	43	67	41	69	80	30
ESCC	Liu F [24]	2013	143/143	60	108/35	−	−	116	27	38	56
	Wang F [25]	2010	30/75	60.48 ± 8.73	45/30	27	48	55	20	39	36
EHCC	Watanabe A [6]	2014	0/80	−	58/22	22	58	59	22	69	11
UUT-UC	Lin WC [26]	2009	0/58	64.6 ± 12.7	30/28	−	−	43	15	12	46
CCA	Xi W [27]	2009	0/148	47.4	0/148	64	84	−	−	35	113
LC	Zou ZQ [12]	2015	0/114	−	82/32	69	45	72	24	−	−
	Nie W [10]	2015	37/37	55	72/41	74	39	65	48	72	41
EC	He X [13]	2016	30/84	53.07 ± 7.5	−	69	15	72	12	11	73
NPC	Hsu HP [28]	2013	0/124	48.6	95/29	38	86	−	−	68	56
BCA	Curmi PA [29]	2000	16/133	57	0/133	79	45	79	45	−	
	Golouh R [30]	2007	0/125	67.9	−	−	−	129	85	99	109
OSCC	Kouzu Y [31]	2006	0/81	−	48/33	29	52	51	30	40	41
Glioma	Dong B [32]	2012	20/68	−	30/38	−	−	−	−	−	−
cSCC	Li X [8]	2015	10/52	−	20/32	47	5	40	12	−	−

N, cases; CRC, colorectal cancer; PDAC, pancreatic ductal adenocarcinoma; HCC, hepatocellular carcinoma; GC, gastric cancer; ESCC, esophageal squamous cell carcinoma; EHCC, extrahepatic cholangiocarcinoma; UUT-UC, upper urinary tract urothelial carcinoma; CCA, cervical carcinoma; LC, lung cancer EC, endometrial carcinoma; NPC, nasopharyngeal carcinoma; BCA, breast carcinoma; OSCC, oral squamous-cell carcinoma; cSCC, cutaneous squamous cell carcinoma.

**Table 2:** Methodology and quality of inclined studies

Source of tumor	Authors	Year	Control/cancer	Country	Research design	Resources of samples	Test method
CRC	Ogino S [15]	2009	0/546	USA	Retrospective	Tumor tissue	IHC
	Tan HT [16]	2011	0/324	Singapore	Retrospective	Tumor tissue	IHC; RT-PCR
	Zheng P [17]	2009	0/149	China	Retrospective	Tumor tissue	IHC
PDAC	Li J [18]	2015	40/87	China	Retrospective	Tumor tissue	IHC; RT-PCR
	Lu Y [14]	2014	0/54	Taiwan	Retrospective	Tumor tissue	RT-PCR
HCC	Yuan RH [20]	2006	21/21	China	Retrospective	Tumor tissue	IHC; RT-PCR
	Gan L [19]	2010	72/120	China	Retrospective	Tumor tissue	IHC; RT-PCR
GC	Ke B [23]	2013	40/40	South Korea	Retrospective	Tumor tissue	IHC; RT-PCR
	Jeon TY [21]	2010	0/226	China	Retrospective	Tumor tissue	IHC; RT-PCR
	Liu X [9]	2015	56/56	China	Retrospective	Tumor tissue	IHC; RT-PCR
	Kang W [22]	2012	0/111	China	Retrospective	Tumor tissue	IHC
ESCC	Liu F [24]	2013	143/143	China	Retrospective	Tumor tissue	IHC
	Wang F [25]	2010	30/75	Japan	Retrospective	Tumor tissue	IHC; RT-PCR
EHCC	Watanabe A [6]	2014	0/80	China	Retrospective	Tumor tissue	IHC
UUT-UC	Lin WC [26]	2009	0/58	China	Retrospective	Tumor tissue	IHC
CCA	Xi W [27]	2009	0/148	China	Retrospective	Tumor tissue	RT-PCR
LC	Zou ZQ [12]	2015	0/114	China	Retrospective	Tumor tissue	IHC
	Nie W [10]	2015	37/37	China	Retrospective	Tumor tissue	IHC; RT-PCR
EC	He X [13]	2016	30/84	Taiwan	Retrospective	Tumor tissue	IHC
NPC	Hsu HP [28]	2013	0/124	France	Retrospective	Tumor tissue	IHC; RT-PCR
BCA	Curmi PA [29]	2000	16/133	Slovenia	Retrospective	Tumor tissue	IHC
	Golouh R [30]	2007	0/125	Japan	Retrospective	Tumor tissue	IHC; RT-PCR
OSCC	Kouzu Y [31]	2006	0/81	China	Retrospective	Tumor tissue	IHC; RT-PCR
Glioma	Dong B [32]	2012	20/68	China	Retrospective	Tumor tissue	IHC; RT-PCR
cSCC	Li X [8]	2015	10/52	China	Retrospective	Tumor tissue	IHC; RT-PCR

CRC, colorectal cancer; PDAC, pancreatic ductal adenocarcinoma; HCC, hepatocellular carcinoma; GC, gastric cancer; ESCC, esophageal squamous cell carcinoma; EHCC, extrahepatic cholangiocarcinoma; UUT-UC, upper urinary tract urothelial carcinoma; CCA, cervical carcinoma; LC, lung cancer EC, endometrial carcinoma; NPC, nasopharyngeal carcinoma; BCA, breast carcinoma; OSCC, oral squamous-cell carcinoma; cSCC, cutaneous squamous cell carcinoma; QUADAS, quality assessment for studies of diagnostic accuracy (maximum score 14); IHC, immunohistochemistry, RT-PCR, real time polymerase chain reaction.

### Heterogeneity assessment of studies

The analysis of fixed effects model showed the value of Chi-square was 17.21 with 24 degree of freedom (*P* = 0.08), and the I-square value was 45.6, which indicated that there was no heterogeneity between the studies. Through reviewing the current literature, we also found that there was a very good clinical homogeneity. In the premise of homogeneity, we used fixed models to combine all indices.

### Comparison of stathmin expression between cancer and normal tissues

**Figure 2:**
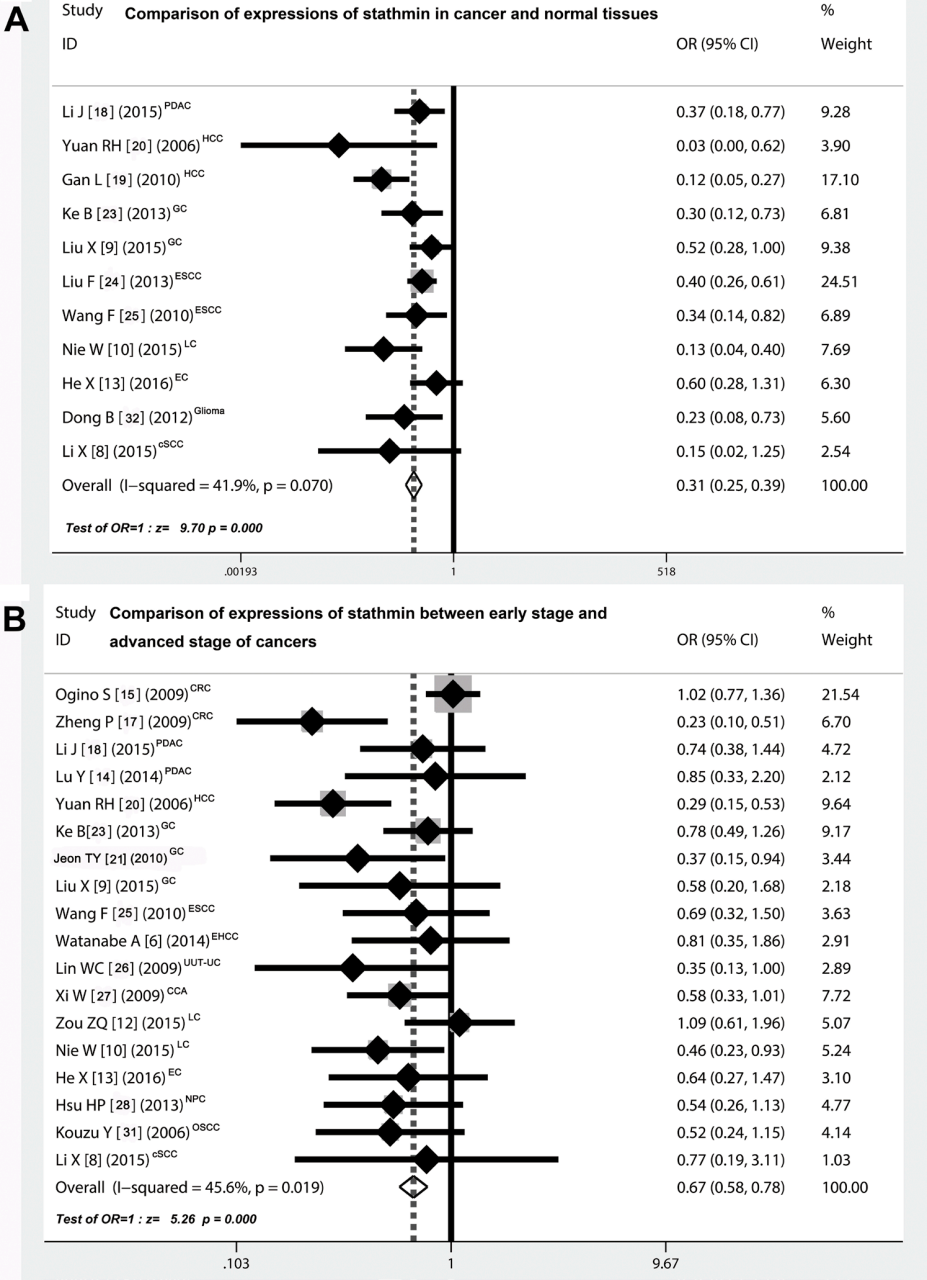
Comparison of expressions of stathmin in cancer and normal tissues (**A**) the overall OR for the combined expressions of stathmin in cancer tissues versus normal tissues was 0.31 (95% CI = 0.25–0.39) in a fixed model (Z = 9.70, *p* < 0.001), suggesting that expression of stathmin was remarkably higher in cancer tissues than in normal tissues; (**B**) the overall OR for the combined expressions of stathmin in early stage versus advanced stage was 0.67 (95% CI = 0.58–0.78) in a random model (Z = 5.26, *p* < 0.001), suggesting that expression of stathmin was significantly higher in advanced stage of cancer patients than in those of early stage; OR, odds ratio; CRC, colorectal cancer; PDAC, pancreatic ductal adenocarcinoma; HCC, hepatocellular carcinoma; GC, gastric cancer; ESCC, esophageal squamous cell carcinoma; EHCC, extrahepatic cholangiocarcinoma; UUT-UC, upper urinary tract urothelial carcinoma; CCA, cervical carcinoma; LC, lung cancer EC, endometrial carcinoma; NPC, nasopharyngeal carcinoma; OSCC, oral squamous-cell carcinoma; cSCC, cutaneous squamous cell carcinoma.

Eleven studies showed the detailed data pertaining to the expressions of stathmin in cancer and normal tissues with control design [8–10, 13, 18–20, 23–25, 32], which included 783 cancer cases and 496 normal controls (Table 3). As shown in Figure 2A, the overall odds ratio (OR) was 0.31 (95% CI = 0.25–0.39) via a fixed model analysis (Z = 9.70, *p* < 0.001), suggesting that expression of stathmin was remarkably higher in cancer tissues than in normal tissues.

**Table 3:** Data extract of stathmin expression in control and cancer patients

Author	Expression of stathmin (positive /all) (N)								Diagnostic test			
	Control (N)	Cancer (N)	Tumor stage (N)		Tumor differentiation (N)		Lymphatic metastasis		TP	FP	FN	TN
			I-II	III-IV	Well to moderate	Poor	Yes	No				
Ogino S [15]	−	546	175/319	122/227	266/499	19/31	−	−	−	−	−	−
Tan HT [16]	−	−	−	−	−	−	−	−	−	−	−	−
Zheng P [17]	−	48/149	9/73	37/69	9/39	11/11	36/70	11/75	−	−	−	−
Li J [18]	11/40	65/87	38/57	27/30	36/54	29/33	39/47	26/40	65	11	22	29
Lu Y [14]	−	32/54	22/39	10/15	20/36	12/18	22/39	10/15	−	−	−	−
Yuan RH [20]	0/21	14/21	17/71	71/85	11/37	77/119	−	−	14	0	7	21
Gan L [19]	7/72	97/120	−	−	−	−	−	−	97	7	23	65
Ke B[23]	8/40	27/40	38/71	95/139	48/85	85/125	85/121	48/89	27	8	13	32
Jeon TY [21]	−	125/226	9/58	15/36	−	−	11/24	13/70	−	−	−	−
Liu X [9]	21/56	40/56	6/14	31/42	16/27	24/29	40/44	3/12	40	21	16	35
Kang W [22]	−	96/114	−	−	34/41	61/69	69/80	26/30	−	−	−	−
Liu F [24]	40/143	101/143	−	−	79/116	22/27	32/38	47/56	101	40	42	103
Wang F [25]	7/30	52/75	14/27	36/48	33/55	19/20	29/39	18/36	52	7	23	23
Watanabe A [6]	−	47/80	11/22	36/58	35/59	14/22	4/11	43/69	−	−	−	−
Lin WC [26]	−	22/58	7/33^*^	15/25^*^	19/43	3/15	8/12	14/46	−	−	−	−
Xi W [27]	−	88/148	27/64	61/84	−	−	18/35	70/113	−	−	−	−
Zou ZQ [12]	−	0/114	52/69	31/45	49/72	18/24	−	−	−	−	−	−
Nie W [10]	4/37	34/37	21/74	24/39	19/65	33/48	38/71	14/42	34	4	3	33
He X [13]	11/30	51/84	38/69	13/15	42/72	9/12	10/11	41/73	51	11	33	19
Hsu HP [28]	−	62/124	12/38	50/86	−		37/68	25/56	−	−	−	−
Curmi PA [29]	−	−	−	−	9/79	17/45	−		−	−	−	−
Golouh R [30]	−	89/125	−	−	45/129	43/85	34/99	52/109	−	−	−	−
Kouzu Y [31]	−	−	12/29	41/52	34/51	19/30	30/40	23/41	−	−	−	−
Dong B [32]	4/20	58/68	−	−	−		−	−	58	4	10	16
Li X [8]	1/10	34/52	29/47	4/5	23/40	8/12	−	−	34	1	18	9

N, cases; TP, true positive; LAC, lung adenocarcinoma; LSCC, lung squamous cell carcinoma; SCLC, small cell lung cancer; FP, false positive; FN, false negative; TN, true negative;

* pT stage.

### Comparison of stathmin expression between early and advanced stage of cancers

Eighteen studies [6, 8–10, 12–15, 17, 18, 20, 21, 23, 25–28, 31] showed the detailed data pertaining to the expressions of stathmin in different clinical stages of cancers. Patients with cancer were divided into two groups according to the tumor-node-metastasis (TNM) classification: I-II stage (early stage) versus III-IV stage (advanced stage), including 1174 cancer cases of early stage and 1100 cases of advanced stage (Table 3). As shown in Figure 2B, the overall OR was 0.67 (95% CI = 0.58–0.78) via a random model analysis (Z = 5.26, *p* < 0.001), suggesting that expression of stathmin was remarkably higher in patients with advanced stage than in those of early stage.

### Comparison of stathmin expression between different differentiated degree of cancer tissues

**Figure 3:**
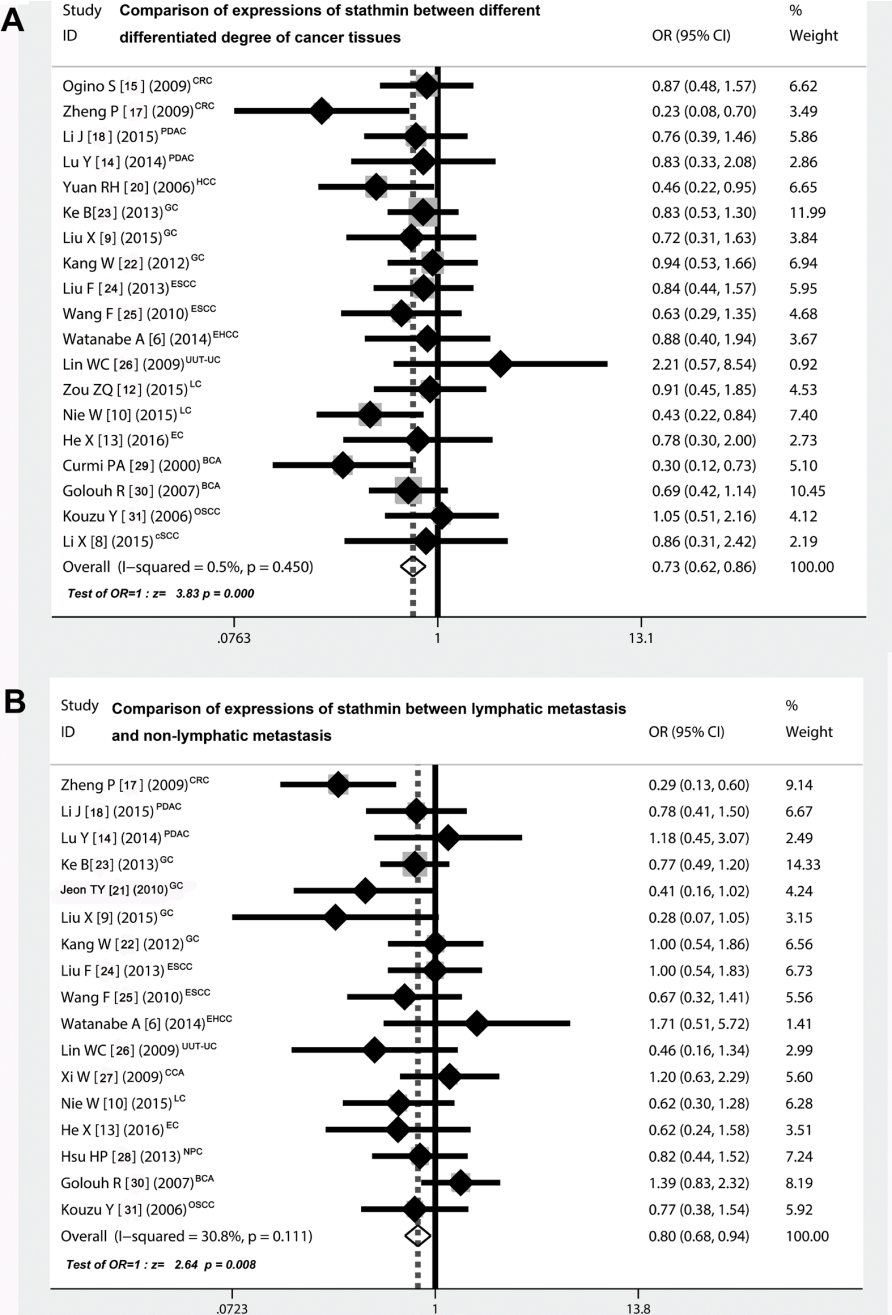
Correlation of expressions of stathmin and different clinical characteristics (**A**) the overall OR for the combined expressions of stathmin in early stage versus advanced stage was 0.73 (95% CI = 0.62–0.86) (Z = 3.83, *p* < 0.001), suggesting that expression of stathmin was remarkably higher in poor differentiated cancer tissues than in well and moderate differentiated cancer tissues; (**B**) the overall OR for the combined expressions of stathmin in lymphatic metastasis versus non-lymphatic metastasis was 0.80 (95% CI = 0.68–0.94) (Z = 2.64, p= 0.008), suggesting that expression of stathmin was remarkably higher in cancer tissues with lymphatic metastasis than those in without lymphatic metastasis; OR, odds ratio; CRC, colorectal cancer; PDAC, pancreatic ductal adenocarcinoma; HCC, hepatocellular carcinoma; GC, gastric cancer; ESCC, esophageal squamous cell carcinoma; EHCC, extrahepatic cholangiocarcinoma; UUT-UC, upper urinary tract urothelial carcinoma; CCA, cervical carcinoma; LC, lung cancer EC, endometrial carcinoma; NPC, nasopharyngeal carcinoma; BCA, breast carcinoma; OSCC, oral squamous-cell carcinoma; cSCC, cutaneous squamous cell carcinoma.

As shown in Figure 3A, nineteen studies [6, 8–10, 12–15, 17, 18, 20, 21, 23, 25–28, 31] showed the detailed data pertaining to the expressions of stathmin in different differentiated degree of cancer tissues (well and moderate *versus* poor differentiated cancer tissues), including 1599 cancer cases of well and moderate differentiation and 775 cases of poor differentiation (Table 3). The overall OR was 0.73 (95% CI = 0.62–0.86) (Z = 3.83, *p* < 0.0001), showing that expression of stathmin was significantly higher in poor differentiated cancer tissues than in those of well and moderate differentiated tissues.

### Comparison of stathmin expression between lymphatic metastasis and non-lymphatic metastasis

As shown in Figure 3B, seventeen studies [6, 9, 10, 13, 14, 17, 18, 21–28, 30, 31] showed the detailed data pertaining to the expressions of stathmin in different status of lymphatic metastasis, including 850 cancer cases of lymphatic metastasis and 972 cases of non-lymphatic metastasis (Table 3). The overall OR was 0.80 (95% CI = 0.68–0.94) (Z = 2.64, *p* = 0.008), suggesting that expression of stathmin was markedly higher in cancer cases with lymphatic metastasis than in those without lymphatic metastasis.

### Analysis of correlation between overexpression of stathmin and overall survival of cancer patients

Four studies reported the correlation between overexpression of stathmin and overall survival of cancer patients [16, 18, 21, 23]. The results showed that patients who exhibited high expressions of stathmin had a significantly shorter post-surgical survival time (27.93 ± 11.54 months) compared with patients who exhibited moderate and low expressions of stathmin (44.81 ± 15.82 months). The t-value was 5.687 and the degree of freedom was three (*P* = 0.01).

### Quality assessment of studies

**Figure 4:**
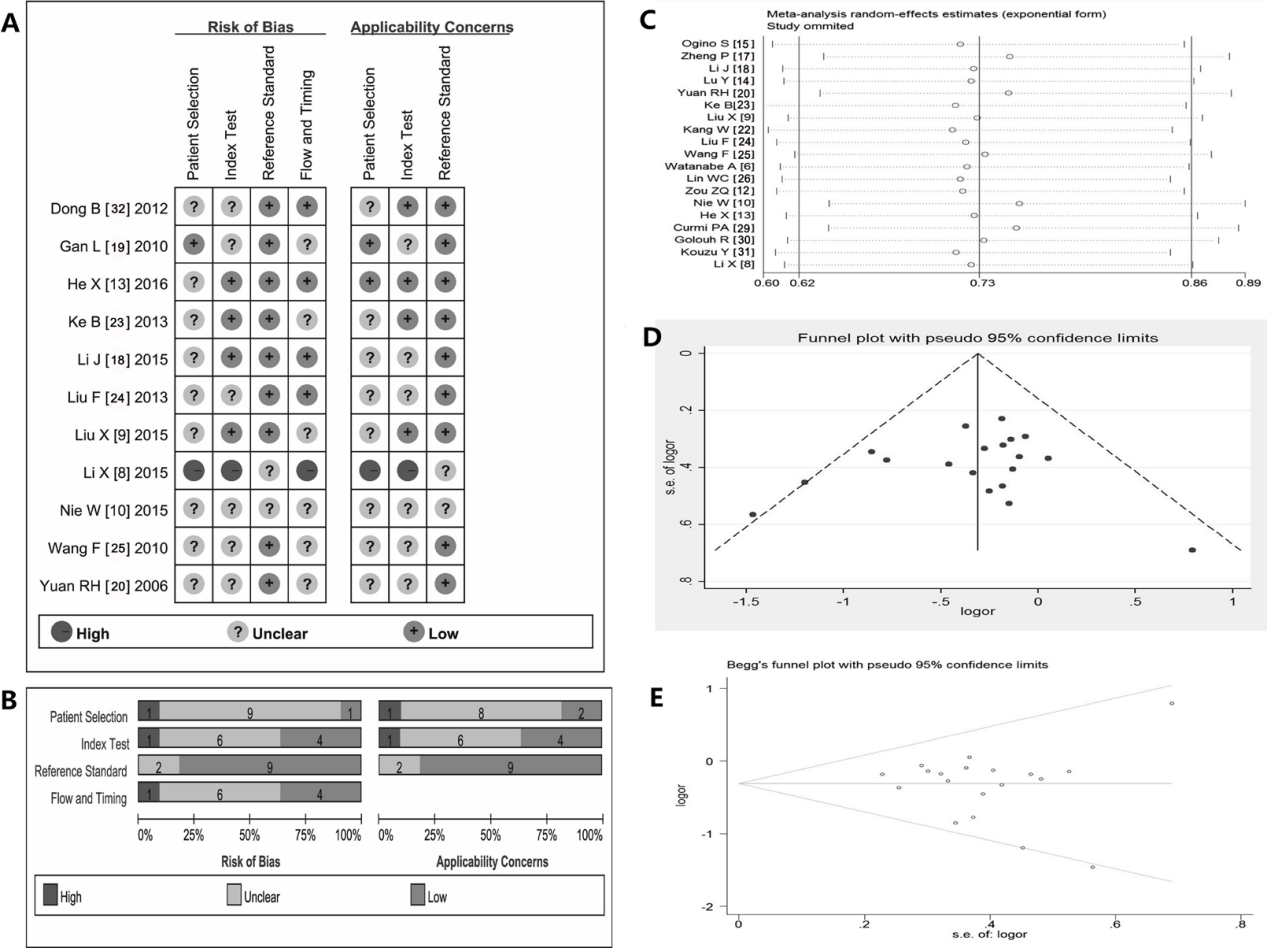
Summary of methodological quality, sensitivity analysis and assessment of publication bias (**A**–**B**) summary on basis of review authors' judgments on seven best differentiating items from QUADAS checklist for each study; (**C**) the exclusion of studies individually did not substantially modify the estimators, with OR values varying between 0.23 and 2.21; (**D**) the shape of the funnel in the funnel plot analysis of publication biases appeared to be approximately symmetrical; (**E**) Begg's test indicated publication biases did not have a significant influence on the results (SD = 28.58, and *p* = 0.421); OR, odds ratio; QUADAS, quality assessment of studies of diagnostic accuracy.

Table 2 showed the principal characteristics on study quality of the 25 studies. All studies were retrospective and all adopted the cancer tissues to detect the expression of stathmin. As shown in Table 4, most of 25 studies had added up to more than 6 stars of scores in evaluating with Newcastle-Ottawa Scale (NOS), which suggested that higher quality studies rigorously controlled for potential confounders [8, 20, 32] and did better with respect to selection of cases and controls [9, 10, 13, 18, 19, 23, 24]. Eleven studies showed the detailed data pertaining to the expressions of stathmin for diagnosis of malignant tumors. Figure 4A and 4B showed summary of methodological quality of included studies according the four best differentiating items from quality assessment of studies of diagnostic accuracy (QUADAS) checklist. Most of studies were evaluated as low risk, and high risk only accounted for less than 10%.

**Table 4:** Assessing the quality of included studies using the Newcastle–Ottawa Scale (NOS)

Authors	Q1	Q2	Q3	Q4	Q5	Q6	Q7	Q8	Q9	Star
Ogino S [15]	⋆	⋆		⋆		⋆	⋆			5
Tan HT [16]	⋆	⋆	⋆	⋆		⋆				5
Zheng P [17]	⋆	⋆	⋆			⋆	⋆	⋆		6
Li J [18]	⋆	⋆		⋆		⋆	⋆	⋆	⋆	7
Lu Y [14]	⋆	⋆	⋆		⋆		⋆			5
Yuan RH [20]	⋆	⋆		⋆	⋆		⋆		⋆	6
Gan L [19]	⋆	⋆	⋆	⋆	⋆	⋆	⋆		⋆	8
Ke B [23]	⋆	⋆			⋆		⋆	⋆		5
Jeon TY [21]	⋆	⋆			⋆		⋆			4
Liu X [9]	⋆	⋆			⋆	⋆	⋆	⋆	⋆	7
Kang W [22]	⋆	⋆			⋆		⋆	⋆		5
Liu F [24]	⋆	⋆	⋆		⋆	⋆	⋆	⋆	⋆	8
Wang F [25]	⋆	⋆	⋆		⋆	⋆			⋆	6
Watanabe A [6]	⋆	⋆			⋆	⋆	⋆	⋆		6
Lin WC [26]	⋆	⋆			⋆	⋆	⋆	⋆		6
Xi W [27]	⋆	⋆			⋆	⋆	⋆	⋆		6
Zou ZQ [12]	⋆	⋆			⋆	⋆	⋆	⋆		6
Nie W [10]	⋆	⋆	⋆		⋆	⋆	⋆	⋆		7
He X [13]	⋆	⋆	⋆		⋆	⋆	⋆	⋆		7
Hsu HP [28]	⋆	⋆			⋆	⋆	⋆	⋆		6
Curmi PA [29]	⋆	⋆			⋆	⋆	⋆	⋆		6
Golouh R [30]	⋆	⋆			⋆	⋆	⋆	⋆		6
Kouzu Y [31]	⋆	⋆			⋆	⋆	⋆	⋆		6
Dong B [32]	⋆	⋆	⋆		⋆	⋆	⋆	⋆		7
Li X [8]	⋆	⋆	⋆		⋆	⋆	⋆	⋆		7

Question 9: Non-response rate

Question 1: Is the case definition adequate?

Question 2: Representativeness of the cases

Question 3: Selection of Controls

Question 4: Definition of Controls

Question 5: Comparability of cases

Question 6: Controls on the basis of the design or analysis

Question 7: Ascertainment of exposure

Question 8: Same method of ascertainment for cases and controls

### Sensitivity analysis and assessment of publication bias

Sensitivity analysis was performed by omitting each study from the estimated pool at every step, the exclusion of any one study individually did not substantially modify the estimators, with OR values vacillating between 0.23 and 2.21 (Figure 4C) [33]. The shape of the funnel appeared to be approximately symmetrical (Figure 4D). In addition, Begg's test indicated the Std. Dev. of Score was 28.58 and Pr > |z| = 0.421. (Figure 4E). Therefore, the funnel plot and Begg test all suggested that publication biases did not have a significant influence on the results [33].

### Sensitivity and specificity of stathmin expression in tumor tissues for the diagnosis of cancers

**Figure 5:**
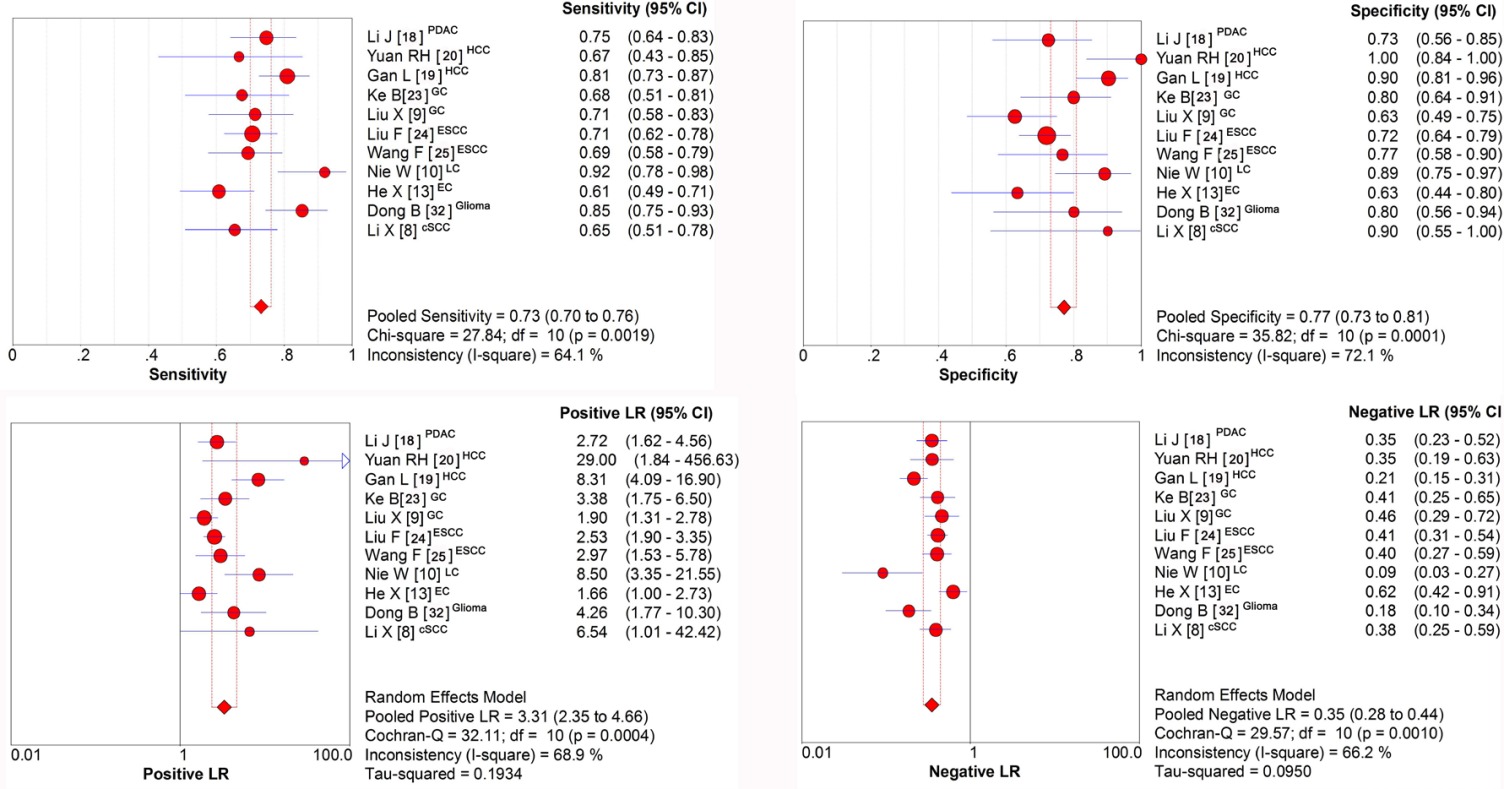
Sensitivity and specificity of stathmin in tissues for the diagnosis of cancer (**A**) sensitivity of stathmin in tissues for the diagnosis of cancer was 0.73 (0.70 to 0.76); (**B**) sensitivity of stathmin in tissues for the diagnosis of cancer was 0.77 (0.73 to 0.81); (**C**) positive likelihood ratio (PLR) was 3.31 (2.35 to 4.66); (**D**) negative likelihood ratio (NLR) was 0.35 (0.28 to 0.44); PDAC, pancreatic ductal adenocarcinoma; HCC, hepatocellular carcinoma; GC, gastric cancer; ESCC, esophageal squamous cell carcinoma; LC, lung cancer EC, endometrial carcinoma; cSCC, cutaneous squamous cell carcinoma.

In order to assess the pooled diagnostic value based on the sensitivity and specificity of stathmin test in tissues for the diagnosis of cancers, we made the forest plots of sensitivity (true positive rate) and 1−specificity (false positive rate) for the 11 research reports [8–10, 13, 18–20, 23–25, 32]. The sensitivity of stathmin in tissues for the diagnosis of cancer was 0.73 (0.70 to 0.76) (Figure 5A) and specificity 0.77 (0.73 to 0.81) (Figure 5B), and the positive likelihood ratio (PLR) was 3.31 (2.35 to 4.66) (Figure 5C) and the negative likelihood ratio (NLR) was 0.35 (0.28 to 0.44) (Figure 5D).

### Diagnostic accuracy of stathmin test in tissues for the diagnosis of cancers

**Figure 6:**
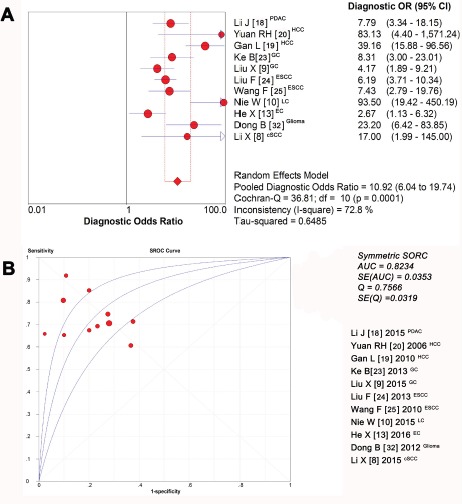
Diagnostic accuracy of stathmin in tissues for the diagnosis of cancer (**A**) the mean DOR was 10.92, indicating that stathmin assay in cancer tissues could be helpful in the diagnosis of malignant tumors; (**B**) the maximum balance joint for sensitivity and specificity was 0.7566 and area under the curve (AUC) was 0.8234; DOR, diagnostic odds ratio; PDAC, pancreatic ductal adenocarcinoma; HCC, hepatocellular carcinoma; GC, gastric cancer; ESCC, esophageal squamous cell carcinoma; LC, lung cancer EC, endometrial carcinoma; cSCC, cutaneous squamous cell carcinoma.

In our present meta-analysis, the mean diagnostic odds ratio (DOR) was 10.92, indicating that stathmin assay in cancer tissues could be helpful in the diagnosis of malignant tumors (Figure 6A). The summary receiver-operator characteristic (SROC) curve help negotiate the balance between sensitivity and specificity of a concrete index in differentiating concrete disease [34]. In our analysis, the maximum balance joint for sensitivity and specificity (the Q-value) was 0.7566. The area under the curve (AUC) was 0.8234 (Figure 6B).

## DISCUSSION

Although stathmin is usually over-expressed in many human cancers, and intimately correlates with the development and progression of malignant tumors [4–11, 13–29, 31, 32]; up to now, we still know relatively little about the role of stathmin in malignant tumors. However, reports on the relationship between abnormal protein expression and cancers, as known, are becoming numerous. The identification of tumor marker has been proposed as a useful strategy to help deeply understanding of human tumorigenesis [33]. In present study, we disclosed the internal connection between abnormal expression of stathmin and malignant tumors and assessed the clinical value of stathmin for discerning malignant tumors.

We used the QUADAS-2 tool to assess included studies, which specially used for evaluating the quality of non-randomized control studies [35]. In addition, Newcastle-Ottawa Scale (NOS) is also a validated technique for assessing the quality of observational and non-randomized studies, which is considered an internationally recognised standard [36]. We employed these two tools to perform the evaluation of study quality and found most of included studies were low risk, suggesting that the conclusion from those studies were reliable and stable. Identifying heterogeneity is important to systematic reviews [37], in our study, we found included studies had a very good clinical homogeneity and statistical analysis showed that the heterogeneity was acceptable. In analysis of sensitivity, we found that the exclusion of any one study did not shake the overall conclusion. In assessment of publication biases, the shape of the funnel plot and Begg's test did not show the statistical significance on publication biases.

In our study, eleven studies compared the expressions of stathmin in cancer with normal tissues [8–10, 13, 18–20, 23–25, 32] and the results suggested that cancer tissues displayed a higher expression of stathmin than normal tissues. Thus, we may infer that higher stathmin expression plays a potentially role in the occurrence and development of cancer. Subsequently, we performed a series of subgroup analysis and found that stathmin displayed a higher expression in advanced cancers, poor differentiated cancers and cancers with lymphatic metastasis, which implied that stathmin intimately involved in tumor cell differentiation, proliferation and invasion of tumors. Metastasis and invasion are the critical factors in the progression of cancer, stathmin has been reported intimately correlating with malignant behavior of tumors [5, 8, 9, 22, 38–41] and affect the survival of tumor patients. In our study, we noticed that patients who exhibited high expressions of stathmin had a significantly shorter post-surgical survival time (27.93 ± 11.54 months) compared with patients who exhibited moderate and low expressions of stathmin (44.81 ± 15.82 months). This phenomena strongly indicated that stathmin may have a potential to become prognostic index for predicting the survival rate of patients with cancer.

In meta-analysis of diagnostic accuracy, we found that the overexpression of stathmin had impressive sensitivity (0.73, 0.70 to 0.76) and specificity (0.77, 0.73 to 0.81) when discriminating the cancer from normal cases, which meant stathmin had a potential to discern cancers. The positive likelihood ratio (PLR) of 3.31 (2.35 to 4.66) suggested that patients with cancer have a nearly 3.31-fold higher chance of being stathmin test positive compared with patients without cancer. This ratio suggested a potential role for stathmin in discerning malignant tumors. Similarly, the negative likelihood ratio (NLR) of 0.35 (0.28 to 0.44) also indicated that low expression of stathmin may help exclude non-cancer individuals. Theoretically, the value of a DOR ranges from zero to infinity, with higher values indicating better discriminatory test performance (higher accuracy) [42]. In our meta-analysis, the value of DOR was 10.92, indicating that the detection of stathmin could be useful to distinguish malignant tumors. The summary measure of test characteristics derived from the SROC curve where sensitivity equalled specificity was 82%, which suggested that the test performance of stathmin in discerning cancer tissues is reasonably good, and it would be a useful tool for the judgement of malignant tumors.

So far, we still know relatively little about how the stathmin regulates tumor proliferation, motility, migration and occurence of metastasis at the molecular level. However, the abnormal expression of stathmin in human tumors has provided a clear possibility for the development of stathmin-dependent molecular diagnosis and targeting therapy. However, we also found some limitations. First, the bias from different tumors may exist because stathmin is highly expressed in many kinds of human cancers. Second, diagnostic review bias may occur because stathmin itself may affect the determination of diagnosis in reports using the clinical course as the gold standard [43]. In the future, it is very crucial to investigate stathmin expression in cases with large samples and multiple clinical centers. Although some deficiencies existed in the studies reviewed, they still provided credible evidence that the stathmin plays an important role in malignant tumors and its detection may be useful for the diagnosis of malignant tumors. In summary, overexpression of stathmin involved in tumor differentiation, lymph node invasion and high TNM stage, suggesting that high expression of stathmin play an important role in malignant tumors. Stathmin had a relatively good sensitivity and specificity suggests that it should be a molecular biomarker for the diagnosis and target for therapy of malignant tumors.

## MATERIALS AND METHODS

### Searching of literature

We searched the published literature from the databases of PubMed, Embase, Cochrane Library and SCI database (from the start of each database up to May 2016). We used the following free text and Medical Subject Heading terms such as “cancer,” “tumor,” “carcinoma,” “tumors,” “neoplasms,” “malignant neoplasms,” “malignant tumors,” “Op18,” “p18,” “p19,” “stathmin 1,” “stathmin”, “metablastin”, “expression,” and “diagnosis.” We also performed hand searches from the references of the articles. If necessary, we contacted with the authors of articles by e-mail for detailed information.

### Inclusion and exclusion criteria of literature

The exclusion criteria: (1) must have showed the data on stathmin expression in malignant tumors; (2) must be case-control or cohort association studies; (3) must have clearly described the control; and (4) the number of cases must be greater than or equal to 50. The exclusion criteria: (1) not original articles such as abstracts, letters, editorials and expert opinions and case reports; (2) did not clearly report sufficient data of stathmin expression; (3) patients who were involved in studies had received chemotherapy, radiotherapy and other drug treatment before taking samples; (4) control group was absent; and (5) non-human studies.

### Extraction procedure of key data from literature

Two researchers extracted the useful data independently. They checked the validity of original data, and contacted the authors of articles if it was necessary. When stathmin expression was tested by different measures in one study, we adopted the results from acknowledged and common measurement. If the paper showed stratum data, we collected all strata data to utilize as full as possible. If two abstracters have disputes on some data, the third abstracter would check the data again, and finally reached consensus through discussion.

### Data of extraction

General information included: authors, countries, publication date, gender and age of patients, type of tumors, testing method of stathmin and study design. Critical data included: case number of different groups, histological classification, tumor node metastases (TNM) classification, tumor differentiation degree, concrete data of stathmin expression (number of true positives, true negatives, false positives, and false negatives).

### Quality assessment of studies

We evaluated the quality of primary studies using the Newcastle-Ottawa Scale (NOS), which uses a star system to evaluate observational studies based on three criteria: participant selection, comparability of study groups and assessment of outcome or exposure [44]. We also used the Quality Assessment of Diagnostic Accuracy Studies (QUADAS -2) tool [35] to assess the quality of diagnostic accuracy. The QUADAS -2 tool uses 14 items to assess risk of bias of study, each item is rated “yes,” “no,” or “unclear” [35], and is phrased such that “yes” indicates low risk of bias.

### Statistical analysis

We conducted a series of analysis according to the standard methods recommended for a meta-analysis of diagnostic test evaluations [45]. The odds ratios (OR) and their 95% confidence intervals (CI) was calculated directly using two different meta-analysis approaches (fixed-effects model and random effects model) according to heterogeneity. We calculated the Chi-square value and I^2^ to evaluate heterogeneity between studies. In the absence of statistically significant heterogeneity, we used the fixed effects method to combine the results. If the heterogeneity existed, we used the random effects method. The overall effect was tested using Z-scores, with significance being set at *p* <0.05. We run sensitivity analysis to evaluate whether single study should affect the overall effects and evaluated publication bias through funnel plots, Begg's and Egger's test respectively. If the data were available, we would combine the following variables of test accuracy: sensitivity; specificity; positive likelihood ratio (PLR); negative likelihood ratio (NLR); and diagnostic odds ratio (DOR), which were derived from summary receiver-operator characteristic (SROC) curve. Statistical analyses were performed using SPSS (SPSS Institute, version 22.0, Chicago, USA), RevMan 5.3.3 (Cochrane Collaboration), Meta DiSc statistical software (Version 1.4, Madrid, Spain), and Stata version 14.0 (Stata Corporation, TX, USA). All the tests were two-sided and the significant level was 0.05.
